# Effect of Antiseptic Mouthwash/Gargling Solutions on SARS-CoV-2 Viral Load: A Randomized Clinical Trial

**DOI:** 10.64898/2026.05.20.26353686

**Published:** 2026-05-22

**Authors:** Sepideh Banava, Allan Radaic, Kamarajan Pachiyappan, Nancy F. Cheng, Yvonne L. Hernandez-Kapila, Stuart A. Gansky

## Abstract

**Background:**

The COVID-19 pandemic has caused significant global mortality. Despite declining infection rates, new variants of SARS-CoV-2 continue to emerge, necessitating new prevention strategies.

**Objective:**

This study aimed to evaluate the effect of four over-the-counter (OTC) antiseptic mouthwash/gargling solutions in the U.S., compared with a distilled water control, on SARS-CoV-2 viral load across multiple oral and oropharyngeal sample types.

**Methods:**

This pilot single-center randomized controlled clinical trial enrolled adults in the San Francisco Bay Area, California, who tested positive for COVID-19. Participants were randomized to distilled water, chlorine dioxide, hydrogen peroxide, cetylpyridinium chloride, and essential oils. Participants were instructed to rinse and gargle four times daily for four weeks using standardized instructions to ensure protocol adherence. Samples were collected on Days 1, 7, and 28 and analyzed using reverse transcription–quantitative polymerase chain reaction (RT-qPCR). The primary outcome was the change in SARS-CoV-2 viral load from baseline to Day 28, assessed using cycle threshold (Ct) values. Secondary outcomes included self-reported clinical symptoms and hospitalization.

**Results:**

Forty-nine participants completed the study. No mouthwash demonstrated a statistically significant reduction in SARS-CoV-2 viral load over time. Cetylpyridinium chloride showed a transient increase in Ct values on Day 7 that was not sustained on Day 28. At baseline, throat swab samples had the lowest Ct values across all sample types. Due to limited subgroup sample sizes for secondary outcome measures, no statistical or moderator analyses were conducted.

**Conclusion:**

Further large-scale randomized trials are needed before recommending antiseptic mouthwashes for SARS-CoV-2 prevention or management.

**Trial Registration:**

ClinicalTrials.gov NCT04409873

## Full Text Availability

The license terms selected by the author(s) for this preprint version do not permit archiving in PMC. The full text is available from the preprint server.

